# Functional Assessment of Corticospinal System Excitability in Karate Athletes

**DOI:** 10.1371/journal.pone.0155998

**Published:** 2016-05-24

**Authors:** Fiorenzo Moscatelli, Giovanni Messina, Anna Valenzano, Vincenzo Monda, Andrea Viggiano, Antonietta Messina, Annamaria Petito, Antonio Ivano Triggiani, Michela Anna Pia Ciliberti, Marcellino Monda, Laura Capranica, Giuseppe Cibelli

**Affiliations:** 1 Department of Clinical and Experimental Medicine, University of Foggia, Foggia, Italy; 2 Department Of Motor, Human and Health Science, University of Rome, "Foro Italico", Rome, Italy; 3 Department of Experimental Medicine, Second University of Naples, Naples, Italy; 4 Department of Medicine and Surgery, University of Salerno, Salerno, Italy; University of Szeged, HUNGARY

## Abstract

**Objectives:**

To investigate the involvement of the primary motor cortex (M1) in the coordination performance of karate athletes through transcranial magnetic stimulation (TMS).

**Methods:**

Thirteen right-handed male karate athletes (25.0±5.0 years) and 13 matched non-athlete controls (26.7±6.2 years) were enrolled. A single-pulse TMS was applied using a figure-eight coil stimulator. Resting motor threshold (rMT) was determined. Surface electromyography was recorded from the first dorsal interosseous muscle. Motor evoked potential (MEP) latencies and amplitudes at rMT, 110%, and 120% of rMT were considered. Functional assessment of the coordination performance was assessed by in-phase (IP) and anti-phase (AP) homolateral hand and foot coordination tasks performed at 80, 120, and 180 bpm.

**Results:**

Compared to controls, athletes showed lower rMT (p<0.01), shorter MEP latency (p<0.01) and higher MEP amplitude (p<0.01), with a significant correlation (r = 0.50, p<0.01) between rMT and MEP latency. Coordination decreased with increasing velocity, and better IP performances emerged compared to AP ones (p<0.001). In general, a high correlation between rMT and coordination tasks was found for both IP and AP conditions.

**Conclusion:**

With respect to controls, karate athletes present a higher corticospinal excitability indicating the presence of an activity-dependent alteration in the balance and interactions between inhibitory and facilitatory circuits determining the final output from the M1. Furthermore, the high correlation between corticospinal excitability and coordination performance could support sport-specific neurophysiological arrangements.

## Introduction

The primary motor cortex (M1) is a complex network of interconnected localized groups of neurons, located in the frontal lobe of the brain,. The role of the M1 is to generate neural impulses that control the execution of movement [1]. Heavily involved in voluntary movements, the M1 shows a high degree of plasticity and adaptation, due to motor learning and practice [2, 3], which determine modifications in the number of synapses, synaptic strength, and topography of stimulation-evoked movement representations [4]. In particular, training induces persistent encoded behaviors within the adult nervous system [5, 6] to allow the precise execution of difficult motor tasks [7, 8].

Karate is considered as one of the most popular martial arts practiced worldwide [9]. It is divided into two competitive disciplines: kata and kumite. Kata consists of prescribed sequences of offensive and defensive techniques and movements, while kumite is a free form of sparring against an opponent. In competitions, kata performers are judged based on specific parameters: technique, rhythm, power, expressiveness of movements, and kime (i.e., short isometric muscle contractions performed at the end of a technique) [10, 11]. The karate fight (i.e., kumite) requires high technical skills (i.e., kick and punch) with precision and high velocity to adequately execute effective attack and defense techniques [12–15]. In addition, technical performance in karate is considerably saturated by cognitive abilities and efficient attentional processes allowing more time for preparation and organization of motor behavior and ensuring quick and correct responses to visuospatial stimuli.

Because it requires a high level of coordination for the precise execution of technical skills in static and dynamic conditions, karate could represent a valuable model to investigate the effects of training on the corticospinal system excitability of athletes [16]. In fact, differences in motor control between expert and novice karate athletes have been observed, mainly attributed to the microstructure of white matter in the cerebellum and M1 [17].

As a non-invasive technique, transcranial magnetic stimulation (TMS) and neuroimaging techniques have been largely used to investigate adaptive changes in human motor cortex [18, 19], contributing to understand how networks in the brain build and optimize the motor programs responsible for coordination of muscle activity involved in complex motor learning [5]. Due to the measurable characteristics of the motor evoked potential (MEP) from peripheral muscles, motor cortex excitability has become a relevant topic in TMS studies [3]. The repetitive practice of simple movements, such as ballistic movements of the digits [18] showed evidence of motor cortex adaptations with similarity to motor learning processes. The improvement in task performance was accompanied by an immediate increase in MEP response, and cross-sectional studies have revealed comparable changes among individuals with varying degrees of motor skill [20]. The motor cortex reorganization is highly dependent on the specific behavioral demands of the training experience. Highly skilled racket players have larger hand motor representation and enhanced MEP amplitudes compared with less proficient players and nonplaying controls [21]. Moreover, highly skilled volleyball players have significantly larger and more overlapping representations of medial deltoid and carpi radialis muscles, than runners [22]. Furthermore, TMS could be suitable for investigating the effect of acute exercise on the excitability of the motor pathway [23]. In particular, augmented amplitudes of MEP have been reported as a result of acute exercise bouts, substantiating the increased neuronal excitability during fatigue [24–27]. Finally, the cortical excitability has been also used as a neurophysiological basis for the inter-limb coordination of young adults [28]. However, no study investigated the chronic effects of training in both MEP and inter-limb coordination performance.

The aim of this study was to examine the differences between karate athletes and non athletes in resting motor threshold (rMT) and MEP responses, and to analyze the relationships between neural activity in M1 and inter-limb coordination performance. Therefore, we hypothesized that athletes might show different cortical excitability in resting condition compared to non athlete counterparts, and that such a difference might be correlated to inter-limb coordination performances, thereby proving that better performance in motor coordination test would reflect higher cortical excitability.

## Materials and Methods

### Participants

Thirteen right-handed male black belt-karate athletes (age: 25.0±5.0 years; height 176.6±6.3 cm; body mass: 78.4±3.6 kg) and 13 age-matched non athlete controls (age: 26.7±6.2 years; height 176.0±7.1 cm; body mass: 80.1±7.1 kg) were recruited. All ssubjects were all right-handed as revealed by the Edimburg inventory test [29]. The karate athletes were members of the local karate team, regularly competing at national and international levels and undergoing a training regimen of at least five 2-hr sessions^.^week^-1^ for the previous 10 years. The control subjects declared to be engaged neither in competitive, nor in amateur sports. The Institutional Ethics Committee of the University of Foggia approved the study. Participants were provided with comprehensive information regarding the possible risks and discomforts by TMS, and were ensured that they were free to withdraw from the study at any time. Furthermore, a medical examination ascertained the absence of psychoactive or vasoactive drugs consumption, or any other contraindication to TMS [30]. All subjects gave their written informed consent before participation.

### TMS and Electromyographic Recordings

To minimize possible circadian influences, measurements were performed between 2:00 and 4:00 p.m., with the subject sitting on an armchair in a quiet room. The motor cortex excitability was evaluated using a 70-mm figure-of-eight coil connected to a Magstim Rapid^2^ (maximum output 2.2 T) Transcranial Magnetic Stimulator (Magstim Company Ltd, UK), placed over the left motor cortex. Surface recording electrodes were placed over the first dorsal interosseous (FDI) muscle. A mechanical arm maintained the handle of the coil tangential to the scalp with the handle pointing backward at 45° away from the midline, while delivering stimulus. The location of stimulation was identified on each subject’s scalp using the SofTaxic navigator system (E.M.S. Italy, www.emsmedical.net). Individual resting motor threshold (rMT) was determined from the left motor cortex, according to a standardized procedure [31]. This threshold has been defined as the minimum stimulation intensity needed to elicit an MEP of at least 50 μV with 50% probability (e.g. 5 out of 10 stimuli) in a fully relaxed muscle [31].

Therefore, to ensure the same relative intensity of stimulation across participants, the stimulation intensity was set at 110–120% of the intensity that elicits rMT, considered as the ‘basic unit of dosing’ [32].

Following TMS, MEP latencies (i.e., the velocity at which the neural signal is propagated from the motor cortex to the muscle) and amplitudes (i.e., the magnitude of corticospinal excitability) at rMT, 110% of rMT (110%rMT), and 120% of rMT (120%rMT) were measured by means of surface electromyography (EMG) recordings (Biopac MP150, BIOPAC Systems, Inc., CA, USA) in the first dorsal interosseus (FDI) muscle of the right hand. Using a classical belly-tendon montage, surface electrodes (1 cm, diameter) were placed in correspondence of the FDI muscle (active electrode) and over the associated joint or tendon (reference electrode), whereas the ground electrode was placed on the dorsal part of the forearm. The magnetic stimulator was connected to the PC, and interfaces with the EMG recording software. The stimulator sends a square wave signal (Trigger) each time it is activated. So on the EMG trace first was shown the trigger and immediately after the muscle response. The latency time was considered the time between the trigger itself (onset of the square wave) and the start of muscle response.

Raw EMG signals were processed and analyzed (Acknowledge software, version 4.1, BIOPAC Systems, Inc., CA, USA) with a high pass filter (cutoff frequency: 10 Hz). For rMT condition, five responses were averaged. For 110%rMT and 120%rMT conditions, ten responses were averaged. We tested the protocol on left cortex for all subjects.

### Interlimb Coordination Performance

Homolateral hand and foot coordination was evaluated by means of a field test [33], which proved to discriminate the effects of training in situational and closed skill sports [34, 35]. Capranica et al. has been validated a field interlimb coordination test in children, adults, and older individuals using the time of correct execution as the main dependent variable [36]. The participants were seated shoeless on a table with elbows and knees flexed at 90 degrees. Observing the spatial and temporal constraints of the movement patterns, they had to perform cyclic flexion and extension movements around the wrist and ankle joints with a 1:1 ratio. Two homolateral coordination modes were tested: in-phase (IP) (i.e., association of hand extension with foot dorsal flexion and hand flexion with foot plantar flexion) and anti-phase (AP) (i.e., association of hand flexion with foot dorsal flexion and hand extension with foot plantar flexion). Each test condition was performed at 3 different frequencies (80, 120, and 180 bpm, respectively) dictated by a metronome for a total duration of 60 s. During the 2-minute rest between test trials, the subjects were allowed to stand. Following 15 seconds of the required metronome pace, a ‘‘ready-go” command indicated the start of a trial. Using a stopwatch, an observer measured the time (s) of correct execution from the beginning of the movement until the subject failed to meet either the spatial and/or the temporal task requirements. To avoid disagreement among observers, a single competent observer scored the inter-limb performances.

### Statistical Analysis

The R Project for Statistical Computing software (version 3.1.0) was used for statistical analyses. Means (M) and standard deviations (SD) were calculated for each of the analyzed variables and statistical significance was set at p ≤ 0.05. The Shapiro-Wilk test was used to verify the normal distribution of variables. T-test was used to ascertain differences between groups for rMT. Analysis of variance (ANOVA) for repeated measure was performed to investigate the differences between groups for MEP latency and amplitude. A 2 (Groups: Athletes, Controls) x 2 (Coordination Mode: IP, AP) x 3 (Execution Frequency: 80, 120, 180 bpm) ANOVA for repeated measures was applied to the time (s) of correct execution of the inter-limb coordination test. If the overall F test was significant, Tukey’s post-hoc comparisons were used. Pearson product-moment correlation was performed to ascertain the relationship between rMT and MEP latency, and between rMT and coordination performances. Cohen’s effect sizes (ES) were also calculated for significant differences. An ES ≤0.2 was considered trivial, from 0.3 to 0.6 small, <1.2 moderate, and >1.2 large, respectively.

## Results

**Fig 1:**
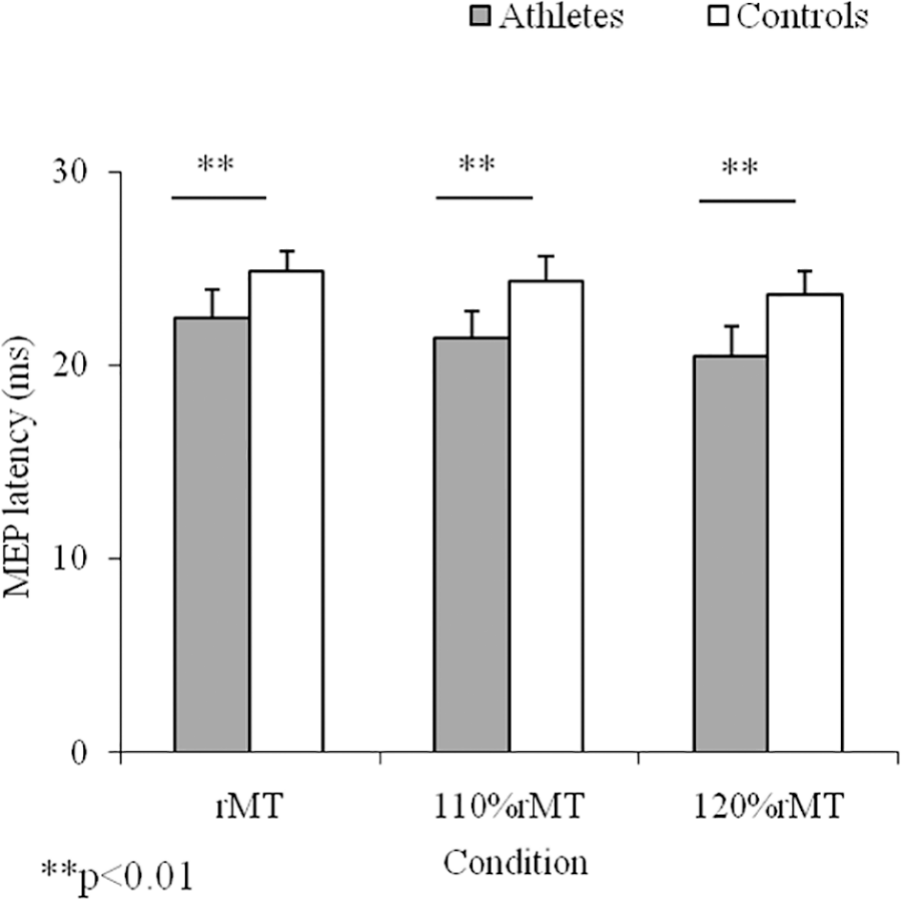
Motor Evoked Potential (MEP) Latency. Means and SDs of MEP latency in the three experimental conditions for karate athletes and controls.

**Fig 2:**
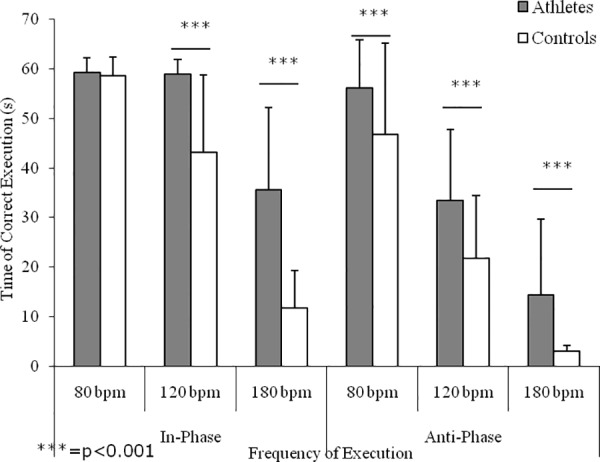
Motor Evoked Potential (MEP) Amplitude. Means and SDs of MEP amplitude in the three experimental conditions for karate athletes and controls.

No discomfort or adverse effects during TMS were noticed or reported. In general, karate athletes showed lower (p**<**0.01; ES = 1.7) rMT (57.3±4.3%) with respect to controls (64.8±4.5%). Shorter MEP latencies (F(5,84) = 14.38; p<0.0001) were observed in karate athletes. For MEP latency, post-hoc analysis maintained differences between groups for rMT (p**<**0.01; ES = 1.8), 110% rMT (p**<** 0.01; ES = 0.73), and 120%rMT (p**<** 0.01; ES = 0.7) conditions (Fig 1). With respect to controls, higher MEP amplitudes (F(3,56) = 146.9; p<0.001) were found in karate athletes. Post-hoc analysis (Fig 2) showed differences between groups only for 110% rMT (p<0.01; ES = 7.1).

**Fig 3:**
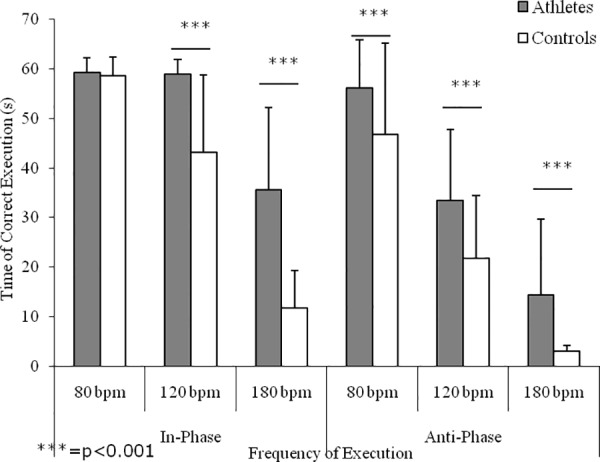
Interlimb coordination performance. Means and SDs of the time of correct execution of homolateral hand and foot synchronized movements in relation to execution mode (i.e., in-phase and antiphase) and velocity of execution (i.e., 80, 120, and 180 bpm) of karate athletes and controls.

Fig 3 shows the interlimb coordination performances in the IP and AP conditions. Main effects emerged for group (F(1,20) = 353.67, p<0.0001), with athletes showing higher values with respect to controls; frequency of execution (F(2,20) = 1593.72, p<0.0001), with higher values at the lower frequencies of execution; and for coordination mode (F(1,20) = 747.05, p<0.0001), with better performances during the IP conditions with respect to AP ones. Significant interactions emerged for frequency of execution x coordination mode (F(2,20) = 85.32, p<0.0001) and for frequency of execution x group (F(2,20) = 57.09, p<0.0001). Except for the time of correct execution at 80 bpm of the IP condition, post hoc analysis showed differences between athletes and controls (ES range: 0.63–4.57).

**Fig 4:**
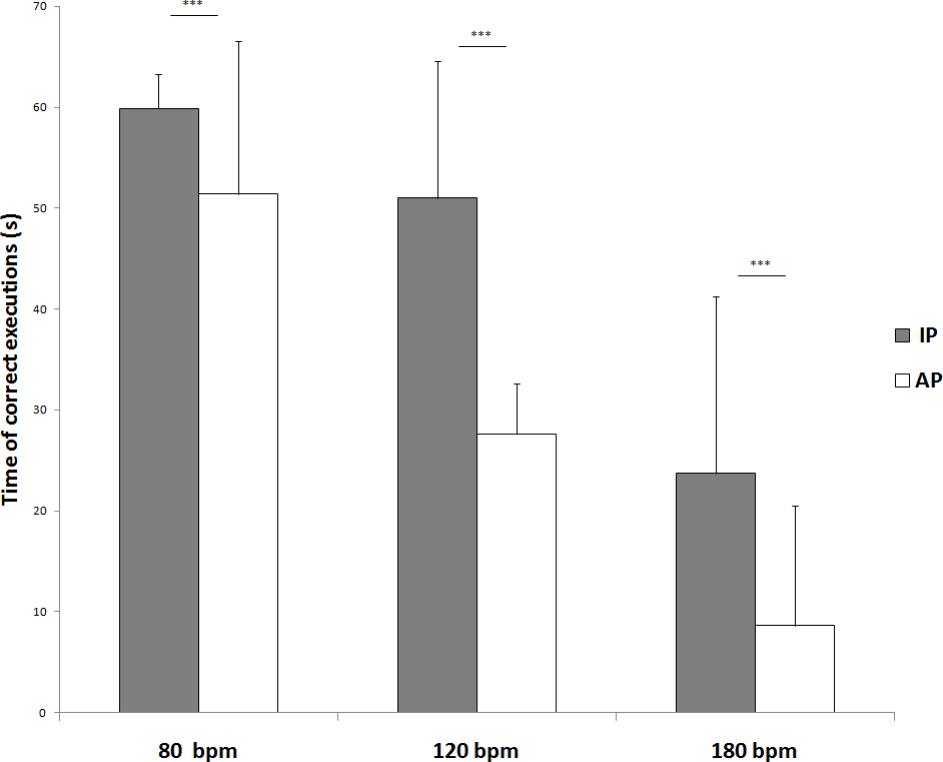
Post hoc analysis for frequency of execution x coordination. Means and SDs of the time of correct execution of homolateral hand and foot synchronized movements in relation to execution mode (i.e., in-phase and antiphase) and velocity of execution (i.e., 80, 120, and 180 bpm).

Post hoc analysis for frequency of execution x coordination mode showed significant differences at 80 bpm IP vs 80 bpm AP (59.9±3.4 seconds vs, 51.4±15.1 seconds; p<0.001), 120 bpm IP vs 120 bpm AP (51.0±13.6 seconds vs 27.6±5.0 seconds; p<0.01) and 180 bpm IP vs 180 bpm AP (23.7±17.5 seconds vs, 8.6±11.9 seconds; p<0.001) (Fig 4).

Whilst a positive correlation (p<0.01) emerged between rMT and MEP latency, negative correlations emerged between rMT and interlimb coordination performances (Table 1).

**Table 1:** Pearson product-moment correlation.

Parameters	Sample size	Pearson's r	p value
rMT vs MEP latency	26	0.5	<0.01
rMT vs IP 80 bpm	26	-0.37	n.s.
rMT vs IP 120 bpm	26	-0.44	<0.05
rMT vs IP 180 bpm	26	-0.57	<0.01
rMT vs AP 80 bpm	26	-0.63	<0.01
rMT vs AP 120 bpm	26	-0.41	<0.01
rMT vs AP 180 bpm	26	-0.72	<0.01

Correlation coefficients and p values for the relationship between rMT (%) and MEP latency (ms), and rMT (%) and Interlimb task.

## Discussion

The main findings of this study highlighted differences between groups in rMT, MEP, and interlimb coordination, and significant correlation between rMT and MEP response, and between rMT and coordination parameters. In particular, the lower rMT, shorter MEP latency and higher MEP amplitude observed in karate athletes support the hypothesis that training determines specific brain organizations to meet specific sport challenges. According to literature [5], in this study karate athletes showed different M1 excitability compared to controls, substantiating the role of acquisition and maintenance of specific motor skills on plastic changes in the controlling neural system. Similarly, a decrease in rMT was shown in subjects trained to produce skilled digit movements on a piano, accompanied by an increase in the corresponding area of digit representation [19]. Furthermore, with respect to controls, subjects trained on skilled ankle [37] or tongue tasks [38] also showed increases in the movement representation area and MEP amplitude. The present results showed differences between groups in MEP amplitude at rMT and 110% rMT only. Whereas at 120% rMT the difference resulted not significant. These results indicate that the differences between trained and untrained subjects are observed only on the values of rMT, by increasing the intensity of stimulation, 110–120% of relative rMT, disappear the differences in MEP amplitude. However increasing the intensity of stimulation athletes showed shorter MEP latency demonstrating more efficiency in central motor conduction time. Thus, in athletes, the velocity at which the neural signal is propagated from the motor cortex to the muscle is higher than non-athletes in each condition.

According to the literature [33, 36, 34, 35], spatial and temporal constraints of synchronized interlimb coordination tasks have been used to investigate the effects of chronic training on the capability of the central nervous system to maintain a stable phase relation between segments with different mechanical characteristics. Independently from training, the present findings confirmed that interlimb coordination performances are function of frequency of execution and coordination mode, with better performances shown on isodirectional tasks and at slower execution frequencies [33, 39, 40]. However, at the lowest velocities of the IP condition karate athletes succeeded in maintaining the synchronized movements for the total duration of the test and showed a significant decrease only at the highest execution frequency, which requires a high level of attentional monitoring. These results are in line with that reported for soccer [34, 41, 42] and basketball [35] players, confirming that the practice of sports with high coordinative demands enhances the efficiency of those executive and attentive control functions involved in complex motor behaviors. Furthermore, in the AP condition karate athletes showed better performances with respect to sedentary counterparts, confirming that chronic training assists players to avoid the spontaneous switch to easier IP movement patterns [33, 36, 34, 35, 41, 42], probably due to its positive effects on executive functions [43] in addition to an enhanced attentional control [44]. Coordinated cyclic movements of the ipsilateral hand and foot are more unstable and inaccurate when they are moved in opposite directions than when they are moved in the same direction [19]. Furthermore, this test succeeded in discriminating coordination performances between karate athletes and non athletes, contributing to the understanding of the effects of training on movement coordination. For the IP condition, a significant difference emerged for frequency of execution, indicating that karate practice can improve even the more natural interlimb coordination. In particular, with increasing velocity of execution, karate athletes did not show any decrement in performance between 80 bpm and 120 bpm, and show lower decrease in performance between 120 bpm and 180 bpm compared to non athletes. This phenomenon reflects the higher coordination of professional karate athletes. Also, in the AP condition, karate athletes always showed significantly better performances than sedentary counterparts at 120 bpm and 180 bpm, indicating that chronic karate practice helps athletes to overrule the easier movement patterns, which spontaneously emerge under stressful conditions or during the acquisition of new skills. This study showed that motor practice seems to induce changes in cortical excitability. The inputs from the environment, and also from motor practice, provide feedback for the motor system to accurately perform motor tasks and are essential for motor learning. However, reduced sensory function results in decreased manual motor function and interferes with the recovery of voluntary movements [45]. Neuroanatomical, imaging, neuromagnetic, and electrophysiological studies have investigated the effect of motor learning. These studies have revealed increased activation of the contralateral primary sensory cortex, supplementary motor area, dorsal premotor cortex, posterior parietal cortex, and secondary sensory cortices bilaterally [46–48]. In fact, changes in corticospinal excitability measured at rest, as in our study, may reflect the altered state of circuits.

The significant negative correlation between rMT and MEP latency and positive correlation between rMT and the coordination tests, suggest that the acquisition of new motor skill and the repetition of complex movement, are associated to a high cortical excitability. Athletes’ changes in MT and MEP values might be the result of plasticity of the motor cortex, due to sport practice which improves cortical excitability. It is commonly accepted role of an increased MEP amplitude in the plasticity response that usually accompanies motor skill learning [49]. In fact as show by Cirillo et al. an increase in MEP amplitude of the target muscle following a simple motor-training intervention is thought to reflect use-dependent plasticity, via long-term potentiation-like mechanisms in cortical circuits [50]. A similar increase in MEP amplitude has been observed following visuomotor tracking in young subjects [51, 52], with these complex tasks expected to rely more heavily on processing within cortical circuits and demand more attentional focus for accurate performance [53].

## Conclusion

The differences found in our study could be caused by better cortical connectivity due to training. In fact the adult brain has the ability to modify its organization (Brain plasticity) through physiological mechanisms such as the repetitions of simple movements [21]. However in a changing environment, brain plasticity enables the nervous system to ensure that proper activation of muscles may be acquired and maintained to serve the behavioural goal, and, recently, a genetic component has been observed for brain plasticity [54]. Therefore, further studies are needed to clarify the nature of the differences emerged in cortical excitability between trained and untrained subjects.
