# Correction: Functional Assessment of Corticospinal System Excitability in Karate Athletes

**DOI:** 10.1371/journal.pone.0159846

**Published:** 2016-07-19

**Authors:** Fiorenzo Moscatelli, Giovanni Messina, Anna Valenzano, Vincenzo Monda, Andrea Viggiano, Antonietta Messina, Annamaria Petito, Antonio Ivano Triggiani, Michela Anna Pia Ciliberti, Marcellino Monda, Laura Capranica, Giuseppe Cibelli

Fig 2 is incorrectly duplicated from Fig 3. The authors have provided a corrected version here.

**Fig 2:**
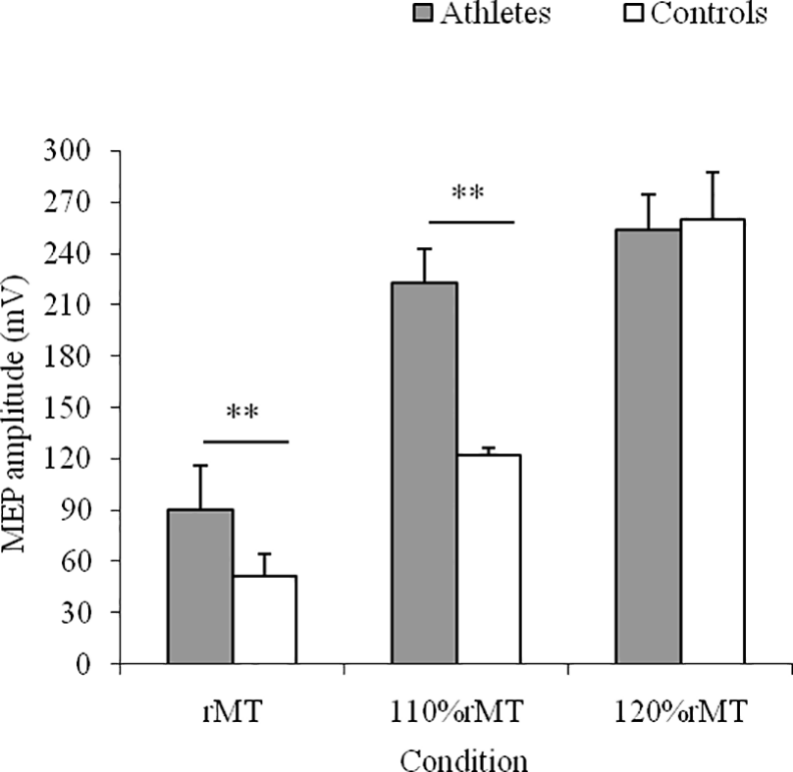
Motor Evoked Potential (MEP) Amplitude. Means and SDs of MEP amplitude in the three experimental conditions for karate athletes and controls.
